# Screening of Tyrosinase, Xanthine Oxidase, and α-Glucosidase Inhibitors from Polygoni Cuspidati Rhizoma et Radix by Ultrafiltration and HPLC Analysis

**DOI:** 10.3390/molecules28104170

**Published:** 2023-05-18

**Authors:** Jing Chen, Qi Huang, Zhuobin He, Guoying Tan, Yuansheng Zou, Juying Xie, Zhengming Qian

**Affiliations:** 1 College of Medical Imaging Laboratory and Rehabilitation, Xiangnan University, Chenzhou 423000, China; chenjing010@126.com; 2 Dongguan HEC Cordyceps R&D Co., Ltd., Dongguan 523850, China; liqilingssz@163.com (Q.H.);

**Keywords:** Polygoni Cuspidati Rhizoma et Radix, enzyme inhibitor, tyrosinase, xanthine oxidase, α-glucosidase, ultrafiltration

## Abstract

Polygoni Cuspidati Rhizoma et Radix (PCR), the rhizome and root of *Polygonum cuspidatum* Sieb. et Zucc., has been used as an herbal medicine for a long time. In this study, the ultrafiltration combined with high performance liquid chromatography (UF-HPLC) method was developed to screen tyrosinase (TYR), α-glucosidase (α-GLU), and xanthine oxidase (XOD) inhibitors from PCR. Firstly, the inhibitory activity of 50% methanol PCR extract on TYR, α-GLU, XOD, and acetylcholinesterase (ACHE) was tested. The extract showed a good inhibition on the enzymes, except for ACHE. Therefore, UF-HPLC experiments were carried out to screen TYR, α-GLU, and XOD inhibitors from PCR extract. Seven potential bioactive components were discovered, including methylgallate (**1**), 1,6-di-O-galloyl-D-glucose (**2**), polydatin-4′-O-D-glucoside (**3**), resveratrol-4′-O-D-glucoside (**4**), polydatin (**5**), malonyl glucoside resveratrol (**6**), and resveratrol-5-O-D-glucoside (**7**). Most of them were found as enzyme inhibitors from PCR for the first time, except polydatin (**5**), which had been reported as an α-GLUI in PCR in the literature. Finally, molecular docking analysis was applied to validate the interactions of these seven potential active components with the enzymes. Compounds **1**–**7** were proven as TYR inhibitors, compounds **2**, **4**–**7** were identified as XOD inhibitors, and compounds **4**–**6** were confirmed as α-GLU inhibitors. In short, the current study provides a good reference for the screening of enzyme inhibitors through UF-HPLC, and provides scientific data for future studies of PCR.

## 1. Introduction

Enzymes are a type of biological substance that maintain the homeostasis and balance of the human body by catalyzing metabolic responses and modulating cells. Enzyme inhibitors can slow the progression of many diseases [1]. α-Glucosidase (α-GLU) is one of the important targets of diabetes, and acarbose, a α-GLU inhibitor, has been widely used to treat diabetes. Xanthine oxidase (XOD) is the key enzyme of gout, and its inhibitor (allopurinol) is a commonly used gout treatment drug. Tyrosinase (TYR) is related to pigmented skin diseases such as chloasma and senile plaques, and acetylcholinesterase (ACHE) is related to Alzheimer’s disease [1,2,3]. Currently, the enzyme inhibitors majorly originate from chemical synthetic, and natural products [1]. Chemical synthetic enzyme inhibitors usually have some side effects; for example, acarbose carries the risk of skin allergy and liver damage, while allopurinol carries the risk of liver and kidney damage [3,4,5,6]. To find safer enzyme inhibitors, screening enzyme inhibitors from natural products has become a research hotspot, and many natural inhibitors, such as huperzine-A, ursolic acid, and resveratrol, have been discovered [1,7]. However, due to the complex composition of natural products, screening enzyme inhibitors from them is extremely difficult [1,8]. The traditional method for screening enzyme inhibitors includes sample extraction, separation, and active testing. These procedures are often laborious and time-consuming. As a result, it is urgent that a rapid and efficient method be developed for screening enzyme inhibitors from natural products. Ultrafiltration coupled with high performance liquid chromatography (UF-HPLC), which combines enzyme affinity, HPLC separation, and identification, is a powerful tool for screening potential bioactive ingredients from complex natural products [3,4,6]. In UF-HPLC analysis, UF is carried out on a semipermeable membrane with a certain molecular weight cut-off which can separate the micro-molecule components and enzyme mixture into two parts. Micro-molecule components bind with enzymes that can be trapped on the membrane, and the unbinding components can directly pass through the membrane. The unbinding sample fractions, which react with enzymes and inactive enzymes, are further detected by HPLC. The sample components with significant reductions in the peak areas of the HPLC chromatograms compared to the inactive enzymes are regarded as potential enzyme inhibitors [9]. The UF-HPLC method overcomes the shortcomings of traditional methods, and has been demonstrated as a simple, rapid, and effective method for screening enzyme inhibitors for natural products [3,6,9].

Polygoni Cuspidati Rhizoma et Radix (PCR) is the rhizome and root of *Polygonum cuspidatum* Sieb. et Zucc. [10]. It is known as “Hu Zhang” in Chinese [10], and is a perennial plant that is primarily grown in Asia and North America. It has been used as an herbal medicine in China, Japan, and Korea for a long time. It is traditionally used to drain dampness, abate jaundice, clear heat, remove toxins, dissipate stasis, relieve pains, suppress cough, and resolve phlegm. Therefore, PCR is frequently used to cure dampness–heat jaundice, turbid stranguries, abnormal vaginal discharge, painful bi disorder caused by wind dampness, swelling abscess, sore and toxin, scald and burn, amenorrhea, abdominal masses, traumatic injuries, and cough caused by lung heat [10]. Recent pharmacological studies have shown that PCR extract has whitening, anti-diabetes, anti-gout, and anti-dementia activities [11,12,13,14]. However, the bioactive components associated with the aforementioned pharmacological activities in PCR extract remain unclear. In order to reveal the active components of whitening, anti-diabetes, anti-gout, and anti-dementia in PCR, the key enzyme inhibitors of these pharmacological activities are screened, such as the anti-diabetes α-GLU inhibitor (α-GLUI), the anti-gout XOD inhibitor (XODI), the whitening TYR inhibitor (TYRI), and the ACHE inhibitor (ACHEI). 

**Figure 1:**
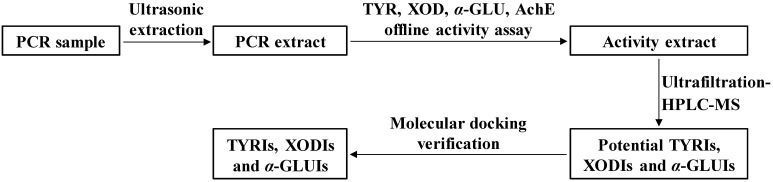
Schematic diagram of the process of screening TYRIs, XODIs, and α-GLUIs from PCR extract.

In the present study, the UF-HPLC method (Figure 1) was developed and applied for the purpose of screening enzyme inhibitors from PCR extract. The enzyme-inhibiting activities of PCR extract were tested by offline assay. The potential enzyme inhibitor of PCR was discovered by UF-HPLC. The potential active components were verified by molecular docking analysis. This study provides the scientific data for future PCR product development and quality standard improvement.

## 2. Results and Discussion

### 2.1. The Inhibitory Activities of PCR Extract on TYR, XOD, α-GLU, and ACHE

The inhibitory activities of PCR extract on TYR, XOD, α-GLU, and ACHE were investigated. It was found that PCR extract inhibited TYR to a degree from 20.24% to 94.21% (104.2 µg/mL–833.3 µg/mL); XOD from 43.93% to 70.75% (34.7 µg/mL–277.8 µg/mL); and α-GLU from 12.43% to 98.49% (0.3 µg/mL–20.8 µg/mL). However, PCR extract had no inhibitory activity on ACHE. The IC_50_ values of the PCR extracts on four enzymes are listed in Table 1. The PCR extract exhibited excellent inhibitory activity on α-GLU (IC_50_ = 0.9 µg/mL), which was superior to that on acarbose (IC_50_ = 176.1 µg/mL). PCR extract also showed good inhibitory activity on TYR (IC_50_ = 220.7 µg/mL), which was comparable to arbutin (IC_50_ = 280.3 µg/mL). The inhibition activity of the PCR extract on XOD (IC_50_ = 63.6 µg/mL) was lower than that on allopurinol (IC_50_ = 0.9 µg/mL). Therefore, PCR extract had inhibitory effects on TYR, XOD, and α-GLU, and the UF-HPLC experiment was further used to screen potential enzyme inhibitors from PCR extract.

### 2.2. Screening of Potential TYRIs, XODIs, and α-GLUIs from PCR Extract by UF-HPLC

**Figure 2:**
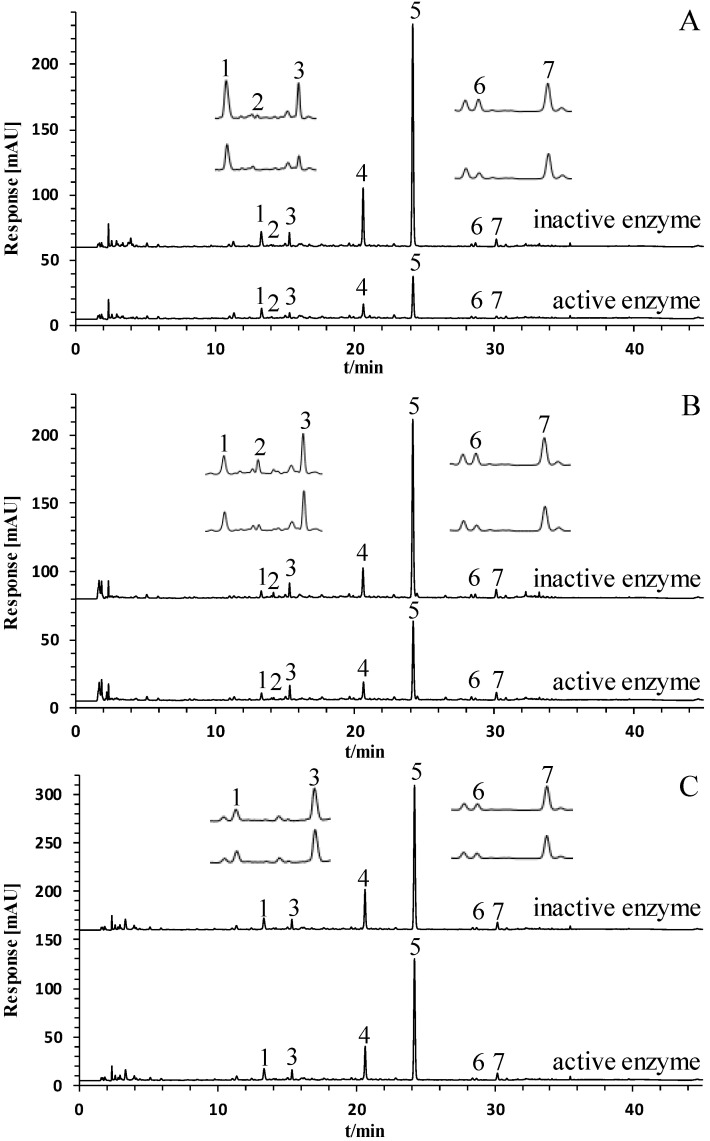
The HPLC chromatograms of PCR extract interacting with three enzymes. (**A**) PCR extract interacting with TYR. (**B**) PCR extract interacting with XOD. (**C**) PCR extract interacting with α-GLU. (1) methylgallate, (2) 1,6-di-O-galloyl-D-glucose, (3) polydatin-4′-O-D-glucoside, (4) resveratrol-4′-O-D-glucoside, (5) polydatin, (6) malonyl glucoside resveratrol, (7) resveratrol-5-O-D-glucoside.

**Figure 3:**
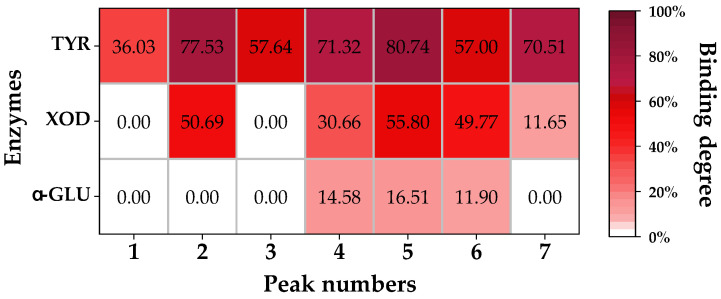
The binding degrees of chromatographic peaks in PCR extract’s interaction with three enzymes.

UF-HPLC is a rapid enzyme inhibitor screening method that has been used in studies on several herbal medicines [3,4,6,9]. By comparing the chromatograms of herbal extracts with active and inactive enzymes, peak areas of active components show a significant decrease. Figure 2 illustrates the results of our UF-HPLC analysis of PCR extracts, demonstrating that the peak areas of seven components were reduced by comparing PCR solution’s interactions with active and inactive enzymes. Figure 3 shows the binding degree of seven chromatographic peaks that interacted with three enzymes. Seven chromatographic peaks (peaks 1–7) were shown to interact with TYR, with binding degrees ranging from 36.03% to 80.74%. Five peaks (peaks 2, 4–7) were shown to interact with XOD, with binding degrees ranging from 11.65% to 55.80%. As for α-GLU, three peaks (peaks 4–6) showed binding degrees ranging from 11.90% to 16.51%. The seven chromatographic peaks mentioned above were proven as potential enzyme inhibitors.

### 2.3. Identification of Potential TYRIs, XODIs, and α-GLUIs from PCR Extract by HPLC-MS

**Figure 4:**
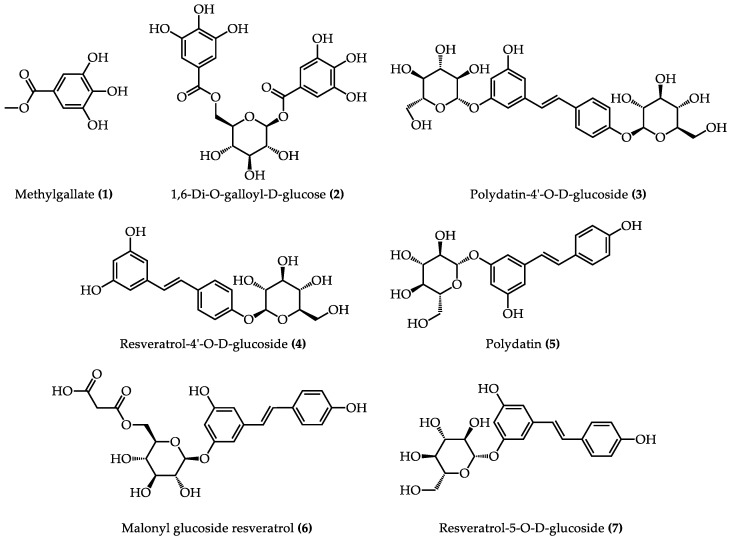
The chemical structures of seven components of PCR extract.

HPLC-MS was applied to identify the seven potential enzyme inhibitors that were screened from PCR extract. MS data for the seven chromatographic peaks are shown in Table 2. Peak 5 was positively identified as a polydatin by comparing the retention time and MS data with the reference compound. The other six chromatographic peaks were tentatively identified as methylgallate (**1**), 1,6-di-O-galloyl-D-glucose (**2**), polydatin-4′-O-D-glucoside (**3**), resveratrol-4′-O-D-glucoside (**4**), malonyl glucoside resveratrol (**6**), and resveratrol-5-O-D-glucoside (**7**) by comparing the MS data with the MassBank database and the literature [15,16,17,18,19]. Figure 4 shows the structures of seven identified components, which include two phenolic components (peaks 1, 2) and five stilbenes (peaks 3–7). The fragmentation pathways of two typical compounds (1,6-di-O-galloyl-D-glucose and polydatin) are shown in Figure S1.

### 2.4. Kinetic Analysis of Enzyme Inhibitors

Seven enzyme inhibitors were found by UF-HPLC analysis in this study, and two of their reference compounds, including methylgallate (compound **1**) and polydatin (compound **5**), can be obtained commercially. The other five reference compounds are difficult to obtain. As described in Section 2.2 and Section 2.3, methylgallate was identified as TYRI, and polydatin was identified as TYRI, XODI, and α-GLUI. The enzyme inhibitory effect and inhibition mode of two compounds were further studied. The results showed that methylgallate had good inhibition on TYR, with IC_50_ value of 102.2 μg/mL, and polydatin showed acceptable inhibition on TYR (IC_50_ = 73.3 μg/mL), XOD (IC_50_ = 830.1 µg/mL), and α-GLU (IC_50_ = 419.9 µg/mL). 

Subsequently, kinetic analysis was conducted using Lineweaver–Burk plots to investigate the inhibition modes on enzymes caused by methylgallate and polydatin. Competitive, noncompetitive, and mixed inhibitions are three common modes in enzyme kinetic analysis. The competitive inhibition manifested as the Michaelis–Menten constant (Km), which increased, and the maximum reaction velocity (Vmax) remained unchanged when the concentration of the inhibitor increased. All of the straight lines almost intersect on the Y axis at a certain point. The noncompetitive inhibition manifested as a decrease in the Vmax, and the Km remained unchanged when the concentration of the inhibitor increased. All of the straight lines almost intersect on the X axis at a certain point. The mixed inhibition manifested as an increase in the Km and a decrease in the Vmax when the concentration of the inhibitor increased. All of the straight lines usually intersect in the quadrants at one point. According to the characteristics of three inhibition modes and combining the results of the Lineweaver–Burk plot (Figure S2), the inhibition mode of methylgallate on TYR was considered to be the noncompetitive type, while the inhibition modes of polydatin on TYR, XOD, and α-GLU were considered to be the mixed type, competitive type, and mixed type, respectively. 

### 2.5. Molecular Docking Analysis

**Figure 5:**
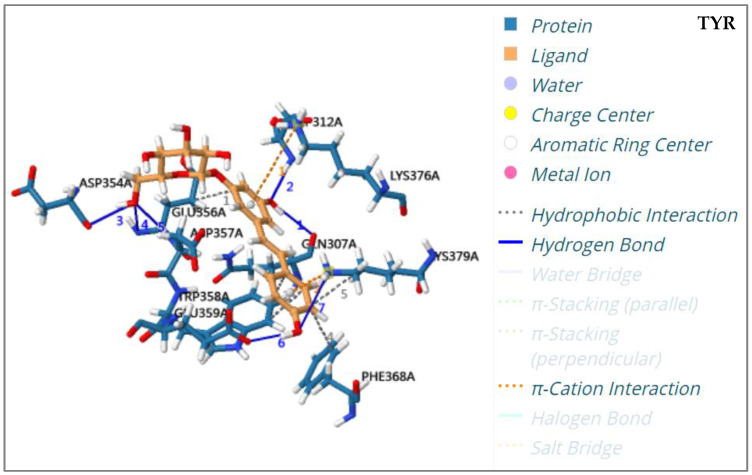

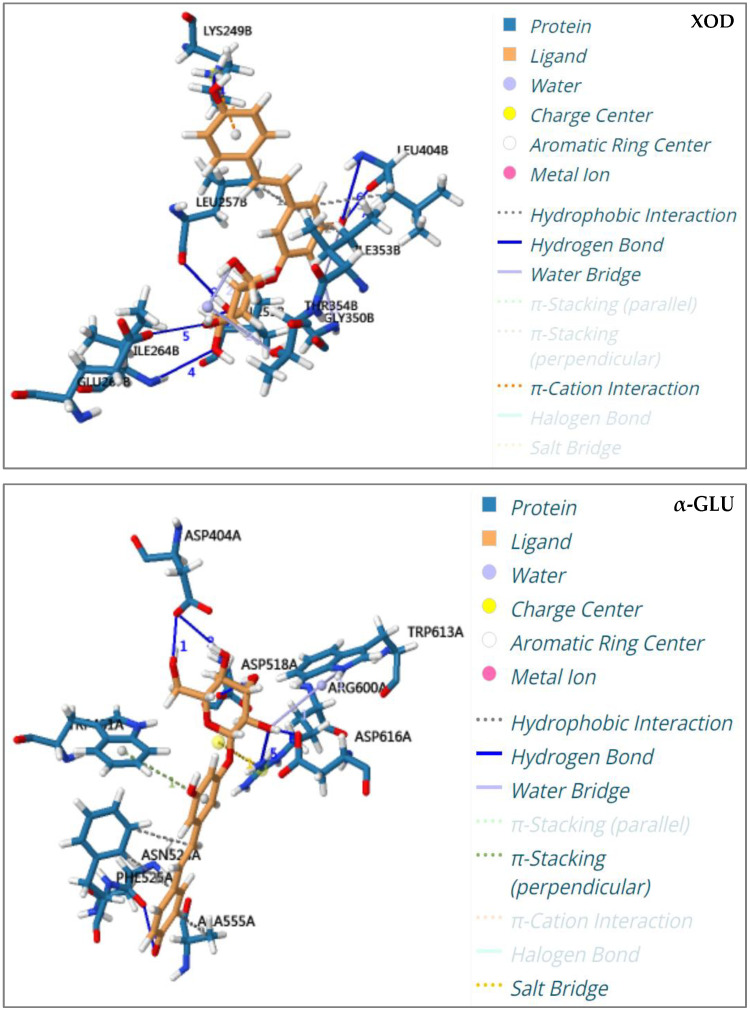
The interaction pattern diagrams of polydatin docked with TYR, XOD, and α-GLU.

Molecular docking is a computer simulation method for investigating the interactions between ligands and receptors, and can be used to predict binding patterns and affinity. In recent years, it has been used to study the bioactivity of enzyme inhibitors from herbal medicine [20,21,22]. In order to verify the activities of seven potential enzyme inhibitors, including methylgallate (**1**), 1,6-di-O-galloyl-D-glucose (**2**), polydatin-4′-O-D-glucoside (**3**), resveratrol-4′-O-D-glucoside (**4**), polydatin (**5**), malonyl glucoside resveratrol (**6**), and resveratrol-5-O-D-glucoside (**7**), that were screened from PCR extract, a molecular docking analysis of these components with TYR, XOD, and α-GLU was carried out. The binding energy and binding residues associated with the interactions between the seven potential enzyme inhibitors and three enzymes are listed in Table 3. It has been reported that the receptor is considered to couple with the ligand when the calculated binding energy score is less than −5.0 Kcal/mol [23]. The results of molecular docking analysis revealed that the binding energy scores of seven potential enzyme inhibitors with related enzymes ranged from −5.215 Kcal/mol to −11.269 Kcal/mol, which were all less than −5.0 Kcal/mol. Further studies found that seven enzyme inhibitors docked with the active centers of the enzyme mainly through hydrogen bonding and hydrophobic interactions. For example, hydrogen bonds were generated between polydatin and enzymes (TYR, XOD, and α-GLU) (Figure 5). Polydatin interacted with TYR, XOD, and α-GLU binding pockets via seven residues (ASP312, ASP354, ASP357, GLN307, GLU356, GLU359, LYS379), six residues (GLU267, ILE264, LEU257, LEU404, LYS249, VAL259), and five residues (ARG600, ASN524, ASP404, ASP518, ASP616), respectively. Hydrophobic interactions also occurred between polydatin and TYR, XOD, and α-GLU binding pockets by interaction with four residues (GLU356, LYS379, PHE368, TRP358), three residues (ILE353, LEU257, LEU404), and two residues (ALA555, PHE525), respectively.

In summary, seven TYRIs (components **1**–**7**), five XODIs (components **2**, **4**–**7**), and three α-GLUIs (components **4**–**6**) were verified by the molecular docking assay. Among them, seven TYRIs (components **1**–**7**), five XODIs (components **2**, **4**–**7**), and two α-GLUIs (components **4**, **6**) were all reported for the first time from PCR. Polydatin (**5**) had been reported as an α-GLUI in PCR by the study referenced in [24].

## 3. Materials and Methods

### 3.1. Chemicals and Materials

Polydatin was acquired from Shanghai Winherb Medical Technology Co., Ltd. (Shanghai, China). Huperzine-A, methylgallate, ρ-nitrophenyl α–D-glucopyranoside (ρNPG), allopurinol, nitrotetrazolium blue chloride (NBT), acetylthiocholine iodide, and 5,5′-dithiobis-(2-nitrobenzoic acid) (DTNB) were obtained from Shanghai Yuanye Biotechnology Co., Ltd. (Shanghai, China). α-glucosidase (α-GLU), acarbose, tyrosine, tyrosinase (TYR), xanthine oxidase (XOD), and acetylcholinesterase (ACHE) were purchased from Sigma-Aldrich (Shanghai, China). Xanthine and HPLC-grade formic acid were supplied by Aladdin Biochemical Technology Co., Ltd. (Shanghai, China). Arbutin was purchased from Shanghai Oli Industrial Co., Ltd. (Shanghai, China). Sodium dodecyl sulfate (SDS) was obtained from Macklin Biochemical Co., Ltd. (Shanghai, China). HPLC-grade acetonitrile was purchased from KRUDE COMPANY, INC. (Los Angeles, CA, USA). The 3 KDa molecular weight cut-off ultrafiltration centrifuge tube (Amicon Ultra-0.5 mL) was bought from Millipore (Darmstadt, Germany). All other analytical-grade chemicals and solvents were obtained from Xilong Scientific Co., Ltd. (Shantou, China). Water was purified by a Milli-Q purification system (Millipore Corp., Billerica, MA, USA).

The Polygoni Cuspidati Rhizoma et Radix (PCR) sample was identified by Dr. Zhengming Qian as the rhizome and root of *Polygonum cuspidatum* Sieb. et Zucc., according to Chinese Pharmacopoeia (2020 Edition Part one). The voucher specimens were deposited at Dongguan HEC Cordyceps R&D Co., Ltd. in Dongguan, China.

### 3.2. Sample Preparations

A quantity of the reference compound polydatin was accurately weighed, then dissolved in 50% methanol to produce a solution containing 80 μg per ml as the reference solution. The reference solution was stored in a dark brown flask at 4 °C, then filtered through a 0.45 μm millipore film prior to HPLC analysis.

The 50 g PCR sample was firstly dried at 50 °C, then crushed and filtered through a No. 3 sieve. A 1.0 g sample of the powder was weighed and ultrasonically extracted (at 380 W power and 37 kHz frequency) by 20 mL 50% methanol solution for 20 min. The supernatant was collected after centrifugation for 10 min (4800× *g*). The supernatant was concentrated and vacuum-dried at 50 °C to obtain the PCR extract powder. PCR extract powder was dissolved in 15% methanol (5 mg/mL) as a sample stock solution. A part of the sample stock solution was used for the UF-HPLC experiment. The rest of the sample stock solution was used for the enzyme inhibition test; it was diluted to a series of concentrations with 5% methanol–PBS solution. The test solution was filtered through a 0.45 μm millipore film prior to HPLC analysis.

### 3.3. Offline Inhibition Test of Four Enzymes

#### 3.3.1. Tyrosinase (TYR) Inhibition Test

TYR inhibition was performed on a 96-well microplate using the method described in the literature, with minor modifications [25]. In the TYR inhibition assay, each well contained 100 µL of the PCR sample and 50 µL of TYR (200 U/mL dissolved in 100 mM of phosphate buffer, pH 6.5). After preincubation at 25 °C for 15 min, 50 μL of tyrosine (5 mM, dissolved in 100 mM of phosphate buffer, pH 6.5) was added to begin the reaction. The reaction was incubated at 25 °C for 50 min. The reaction solution was tested at 475 nm. The control was tested with the same reaction system, but the PCR sample solution was replaced by 100 mM of phosphate buffer (pH 6.5). Arbutin was used as a reference inhibitor in the TYR inhibition assay. The results are expressed as percentages of inhibition, which were calculated by Equation (1).

Inhibition (%) = [(Abs_control_ − Abs_sample_)/Abs_control_] × 100% (1)

#### 3.3.2. Xanthine Oxidase (XOD) Inhibition Test

XOD inhibition was performed on a 96-well microplate using the method described in the literature, with minor modifications [26]. In the XOD inhibition assay, each well contained 30 μL of the PCR sample, 30 μL of XOD (0.05 U/mL, dissolved in 200 mM of phosphate buffer, pH 8.5), and 15 μL of NBT (2 mg/mL, dissolved in 200 mM of phosphate buffer, pH 8.5). After preincubation at 37 °C for 15 min, 90 μL of xanthin (0.5 mM/mL, dissolved in 200 mM of phosphate buffer, pH 8.5) was added to begin the reaction, which was incubated at 37 °C for 30 min. The reaction was stopped by adding 15 µL of SDS (10%). The reaction solution was tested at 560 nm. The control was tested with the same reaction system, but the PCR sample solution was replaced by 200 mM of phosphate buffer (pH 8.5). Allopurinol was used as a reference inhibitor in the XOD inhibition assay. The results are expressed as percentages of inhibition, which were calculated by Equation (1).

#### 3.3.3. α-Glucosidase (α-GLU) Inhibition Test

*α*-GLU inhibition was performed on a 96-well microplate using a modified method described by Talha et al. [27]. In the *α*-GLU inhibition assay, each well contained 40 μL of the PCR sample and 40 μL of the α-GLU solution (0.15 U/mL, dissolved in 100 mM of phosphate buffer, pH 6.8). After preincubation at 37 °C for 15 min, 80 μL of ρNPG (0.5 mg/mL, dissolved in 100 mM of phosphate buffer, pH 6.8) was added to begin the reaction, which was incubated at 37 °C for 30 min. The reaction was stopped with 40 μL of sodium carbonate (1.0 mol/L). The reaction solution was measured at 405 nm. The control was tested with the same reaction system, but the PCR sample solution was replaced by 100 mM of phosphate buffer (pH 6.8). Acarbose was used as a reference inhibitor in the *α*-GLU inhibition assay. The results are expressed as percentages of inhibition, which were calculated by Equation (1).

#### 3.3.4. Acetylcholinesterase (AChE) Inhibition Test

AChE inhibition was performed on a 96-well microplate using the method described in the literature, with minor modifications [28]. In the AChE inhibition assay, each well contained 20 µL of the PCR sample, 20 µL of AChE (2 U/mL dissolved in 200 mM of phosphate buffer, pH 8.0), and 50 µL of DTNB (1.6 mM dissolved in 200 mM of phosphate buffer, pH 8.0). After preincubation at 37 °C for 15 min, 20 μL of acetylthiocholine iodide (4.0 mM, dissolved in 200 mM of phosphate buffer, pH 8.0) was added to begin the reaction, which was incubated at 37 °C for 20 min. The reaction was stopped by 40 µL of SDS (3%), and the reaction solution was measured at 405 nm. The control was tested with the same reaction system, but the PCR sample solution was replaced by 200 mM of phosphate buffer (pH 8.0). Huperzine-A was used as a reference inhibitor in the AChE inhibition assay. The results are expressed as percentages of inhibition, which were calculated by Equation (1).

### 3.4. Screening of Potential Enzyme Inhibitors from PCR by UF-HPLC

#### 3.4.1. UF Conditions

The UF experiment was performed according to the method described in the literature, with slight modifications [4]. In the experimental group, 200 µL of the PCR extraction (5 mg/mL) was mixed with 200 µL of 3 enzyme solutions (200 U/mL of TYR, 4 U/mL of XOD, and 6 U/mL of α-GLU, respectively). The mixtures were incubated at 37 °C for 60 min, and then 200 μL of each of them was transferred to a 3 KDa molecular weight cut-off ultrafiltration centrifuge tube to separate the unbound compounds from the enzyme–ligand complexes by centrifugation (9400× *g*, 30 min). Finally, the ultrafiltration filter was washed three times with 5% methanol–PBS under centrifugal conditions (9400× *g*, 30 min) to completely remove any unbound components. The concentration and pH of the PBS used for the different enzymes were the same as for the offline inhibition tests.

In the control group, inactive enzymes (200 U/mL of TYR, 4 U/mL of XOD, and 6 U/mL of α-GLU, boiled in water for 15 min) were used to replace the active enzymes, and the same operation mentioned above was performed. Finally, the unbound component solutions of the experimental and control groups were analyzed by HPLC. The binding degree of each component was calculated through Equation (2), where P1 is the peak area of components in the control group (interacting with inactive TYR, XOD, and α-GLU enzymes) and P2 is the peak area of components in the experimental group (interacting with active TYR, XOD, and α-GLU enzymes) in HPLC chromatograms.

Binding degree = (1 − P2/P1) × 100%(2)

#### 3.4.2. HPLC-MS Condition

HPLC condition: An Agilent 1260 high-performance liquid chromatography system (Agilent Technologies, Santa Clara, CA, USA), equipped with a quaternary pump system, an auto-sampler, a column oven, and a diode array detector, was employed in the current experiment. The sample was separated on an Agilent Poroshell 120 EC-C18 column (100 mm × 4.6 mm, 2.7 μm). The column temperature was set at 30 °C. The mobile phase was 0.1% aqueous formic acid (A) and acetonitrile (B), with a gradient elution procedure of 5% B (0 min), 5% B (3 min), 20% B (23 min), 24% B (28 min), 60% B (32 min), and 100% B (38 min). The flow rate was 0.6 mL/min. The detection wavelength was set at 290 nm, and the injection volume was 5.0 µL.

MS condition: The Agilent 6530 mass spectrometer system (Agilent Technologies, Santa Clara, CA, USA), with an electron spray ionization (ESI) source, was used in both positive and negative scanning modes from 50–1000 *m*/*z*. The electrospray ionization mass spectrometer conditions were as follows: the capillary voltage was set at 3500 V (+/−); the collision-induced dissociation voltage was set at 120 V; the drying gas (N_2_) flow rate was 8 L/min; the drying gas temperature was set at 350 °C; the nebulizer pressure was 35 psi; and the sheath gas flow rate was 11 L/min.

### 3.5. Kinetic Analysis

In this work, Lineweaver–Burk was used to investigate the inhibition type of methylgallate and polydatin on enzymes. For TYR, the concentration of the enzyme was kept constant at 200 U/mL, and various concentrations of L-tyrosine (1.0~5.0 mM) were prepared. Various concentrations of methylgallate (0.0~1.3 mM) and polydatin (0.0~0.4 mM) were also provided. For XOD, the concentration of the enzyme was kept constant at 0.05 U/mL, and various concentrations of xanthin (0.06~0.50 mM) were prepared. Various concentrations of polydatin (0.0~0.5 mM) were also provided. For α-GLU, the concentration of the enzyme was kept constant at 0.15 U/mL, and various concentrations of ρNPG (0.8~5.0 mM) were prepared. Various concentrations of polydatin (0.0~3.6 mM) were also provided. GraphPad Prism 5.0 software (USA) was used for the calculation.

### 3.6. Molecular Docking Condition

In the molecular docking analysis, Schrodinger software (Maestro 12.8) was used to verify the binding potency of the components to TYR, XOD, and α-GLU. In this process, the structural information of seven components (methyl gallate, 1,6-di-O-galloyl-D-glucose, polydatin-4′-O-D-glucoside, resveratrol-4′-O-D-glucoside, polydatin, malonyl glucoside resveratrol, and resveratrol-5-O-D-glucoside) were obtained from the PubChem platform (https://pubchem.ncbi.nlm.nih.gov/, accessed on 16 March 2023), and the protein crystal structures of TYR (PDB ID = 2Y9X) [29], XOD (PDB ID = 1FIQ) [30], and α-GLU (PDB ID = 5NN8) [4] were downloaded from the Research Collaboratory for Structural Bioinformatics Protein Data Bank (RCSB PDB) (http://www.rcsb.org, accessed on 16 March 2023). The compound structures and protein crystals were imported into the Schrodinger software for pretreatment. The chemical structures of the compounds were all produced with minimal energy. All unnecessary ligands and water molecules were removed from the protein crystals, and hydrogen atoms were added. Other operation parameters were set as default. Finally, docking scores were used to assess the binding capacities of compounds and proteins. Bond residues with interactions were recorded simultaneously.

## 4. Conclusions

PCR is a traditional herbal medicine with a long history of medicinal use. It has multiple pharmacological activities and extensive clinical applications; thus, it possesses a great deal of development value and many application prospects. In this study, seven enzyme inhibitors were discovered from PCR through the UF-HPLC method, including methylgallate (**1**), 1,6-di-O-galloyl-D-glucose (**2**), polydatin-4′-O-D-glucoside (**3**), resveratrol-4′-O-D-glucoside (**4**), polydatin (**5**), malonyl glucoside resveratrol (**6**), and resveratrol-5-O-D-glucoside (**7**). Most of those seven components were found as enzyme inhibitors in PCR for the first time, although polydatin (**5**) has been reported as an α-GLUI in PCR in the literature. The aforementioned findings revealed the concrete bioactive components of the anti-diabetes, anti-gout, and whitening activities of PCR, which will be helpful in the development of PCR products for whitening, anti-diabetes, and anti-gout effects. This also provides scientific evidence for the improvement of the quality evaluation of PCR and its related products.

## Figures and Tables

**Table 1:** IC_50_ values **(µg/mL)** of enzyme inhibitors with TYR, XOD, α-GLU, and ACHE.

Sample	TYR	XOD	α-GLU	ACHE
PCR extract	220.7 ± 2.26	63.6 ± 3.02	0.9 ± 0.10	None
Reference inhibitors	280.3 ± 4.95 (Arbutin)	0.9 ± 0.02 (Allopurinol)	176.1 ± 32.24 (Acarbose)	0.2 ± 0.01 (Huperzine-A)

**Table 2:** MS data of seven components of PCR extract.

NO.	Compound Name	Retention Time (min)	Molecular Formula	Precursor Ion (*m*/*z*)	Fragmentations (*m*/*z*)
1	Methylgallate [15]	13.332	C_8_H_8_O_5_	185.0438 [M + H]^+^	153.0169, 126.0302, 107.0153, 79.0176
2	1,6-Di-O-galloyl-D-glucose [16]	14.191	C_20_H_20_O_14_	483.1769 [M − H]^−^	313.1380, 271.1175, 169.0700, 125.0735
3	Polydatin-4′-O-D-glucoside [17]	15.339	C_26_H_32_O_13_	551.2803 [M − H]^−^	389.2102, 227.1371
4	Resveratrol-4′-O-D-glucoside [17]	20.618	C_20_H_22_O_8_	435.2293 [M − H + FA]	389.2119, 227.1384
5	Polydatin [17,18]	24.178	C_20_H_22_O_8_	435.2239 [M − H + FA]^−^	389.2134, 227.1383, 185.1208
6	Malonyl glucoside resveratrol [19]	28.677	C_23_H_24_O_11_	475.2210 [M − H]^−^	431.2253, 227.1375
7	Resveratrol-5-O-D-glucoside [18]	30.171	C_20_H_22_O_8_	389.2108 [M − H]^−^	227.1368, 185.1186, 143.1006

**Table 3:** The molecular docking analysis results of seven potential enzyme inhibitors of PCR extract.

Components	Enzyme	Docking Score (Kcal/mol)	Amino Acid Residues	Hydrogen Bonds
Methylgallate (**1**)	TYR	−6.036	ASP312, GLN307, LYS379, TRP358, TYR311	ASP312, GLN307, LYS379
1,6-Di-O-galloyl-D-glucose (**2**)	TYR	−6.951	ASP312, ASP353, ASP357, GLU335, GLU356, LYS376, LYS379, THR308, TRP358	ASP312, ASP353, ASP357, GLU335, GLU356, LYS379, THR308
	XOD	−11.901	ALA346, ALA338, ARG426, ASN261, ASN351, ASP360, GLU263, GLY260, LYS422, TRP336, THR354, SER347, VAL259, VAL345	ALA338, ARG426, ASN261, ASN351, ASP360, GLU263, GLY260, LYS422, TRP336, THR354, SER347, VAL259, VAL345
Polydatin-4′-O-D-glucoside (**3**)	TYR	−6.569	ALA220, ARG268, GLU226, GLY223, LEU265, PHE264, THR261, TYR201	ARG268, GLU226, GLY223, PHE264, THR261, TYR201
Resveratrol-4′-O-D-glucoside (**4**)	TYR	−5.215	ASP312, GLU356, GLN307, GLH356, LYS372, TRP358	ASP312, GLU356, GLN307, GLH356, LYS372
	XOD	−9.189	ALA255, ASN261, GLU254, GLY260, ILE353, LEU257, LYS249, LYS256, THR354, VAL259	ALA255, ASN261, GLU254, GLY260, LYS256, THR354, VAL259
	α-GLU	−6.353	ARG281, ARG600, ASN524, ASP282, ASP404, ASP616, HIS674, PHE525, SER523, TRP516, TRP613	ARG281, ARG600, ASN524, ASP282, ASP404, ASP616, HIS674, PHE525, SER523
Polydatin (**5**)	TYR	−6.126	ASP312, ASP354, ASP357, GLN307, GLU356, GLU359, LYS376, LYS379, PHE368, TRP358	ASP312, ASP354, ASP357, GLN307, GLU356, GLU359, LYS379
	XOD	−11.269	GLU267, GLY350, ILE264, ILE353, LEU257, LEU404, LYS249, THR354, VAL259	GLU267, ILE264, LEU257, LEU404, LYS249, VAL259,
	α-GLU	−5.420	ALA555, ARG600, ASN524, ASP404, ASP518, ASP616, PHE525, TRP481, TRP613	ARG600, ASN524, ASP404, ASP518, ASP616
Malonyl glucoside resveratrol (**6**)	TYR	−5.651	ALA286, ARG268, SER282, HIS244, HIS263, PHE264, VAL283	ARG268, SER282
	XOD	−6.415	ALA338, ARG426, ASN351, ASP360, ILE358, LYS422, LYS433, PHE337, SER359, TRP336	ASN351, ASP360, LYS433, SER359
	α-GLU	−5.651	ALA284, ASP282, LEU405, LEU650, PHE649, TRP376, TRP481, TRP516, TRP613, TRP618	ALA284, ASP282
Resveratrol-5-O-D-glucoside (**7**)	TYR	−6.281	ALA246, ALA286, ASN260, HIS244, HIS263, GLU322, PHE264, VAL248, VAL283	ALA246, ASN260, HIS263, GLU322, VAL248
	XOD	−11.625	GLU254, GLU267, GLY350, ILE353, LEU257, LEU398, LEU404, LYS256, THR354	GLU254, GLU267, LEU404, LYS256, THR354

## Data Availability

The data presented in this study are contained within the article.

## Supplementary Materials

The following supporting information can be downloaded at: https://www.mdpi.com/article/10.3390/molecules28104170/s1, Figure S1: Fragmentation pathway of 1,6-di-O-galloyl-D-glucose and polydatin; Figure S2: Lineweaver-Burk plots of methylgallate and polydatin on the activity of three enzymes.

## Sample Availability

Samples of compounds **1** and **5** are available from the authors.
