# Supplementary material for: Screening of Tyrosinase, Xanthine Oxidase, and α-Glucosidase Inhibitors from Polygoni Cuspidati Rhizoma et Radix by Ultrafiltration and HPLC Analysis
Source: Molecules. 2023 May 18;28(10):4170. doi: 10.3390/molecules28104170 (PMC10221946; doi:10.3390/molecules28104170)
Supplement: Supplementary file 1 [file molecules-28-04170-s001.zip › molecules-2316611-supplementary.pdf]

**1,6-di-O-galloyl-D-glucose**

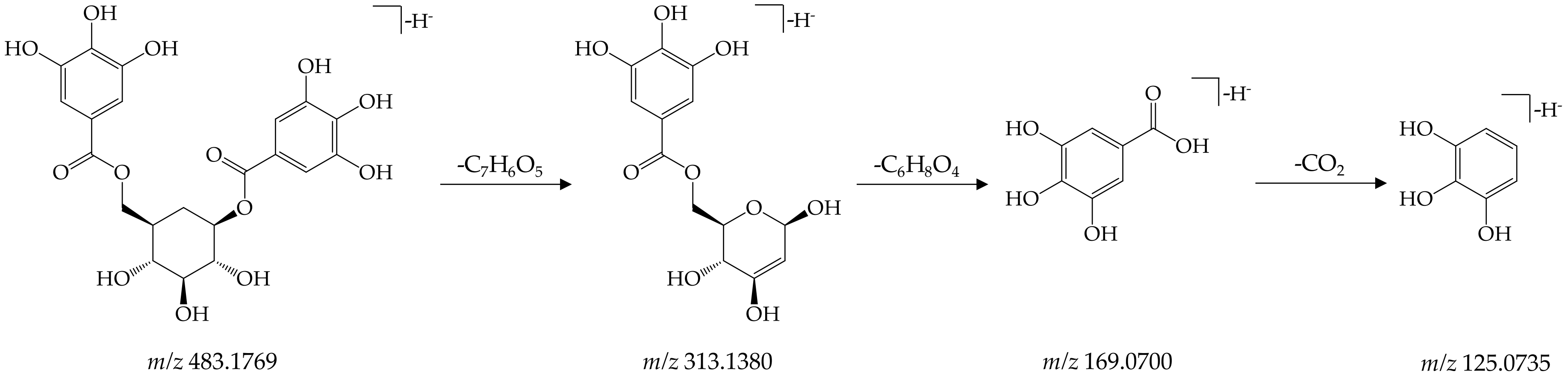


**polydatin**

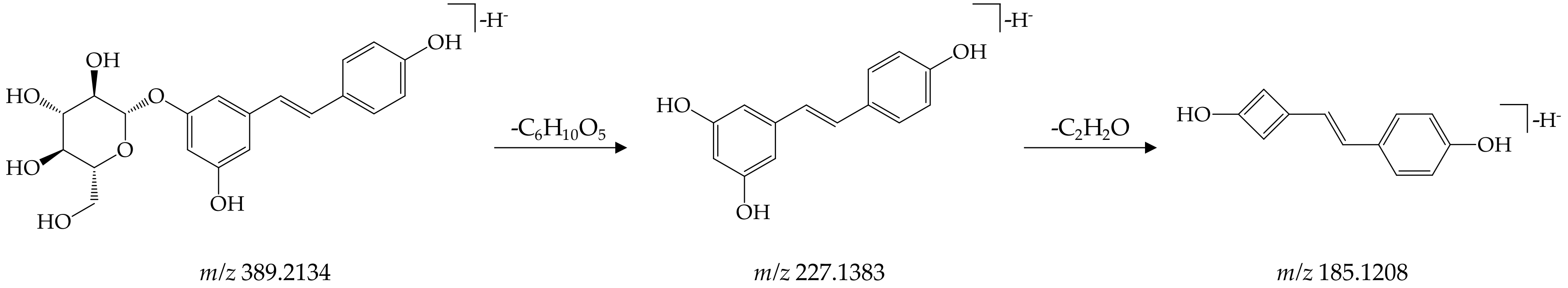


**Figure S1** Fragmentation pathway of 1,6-di-O-galloyl-D-glucose and polydatin

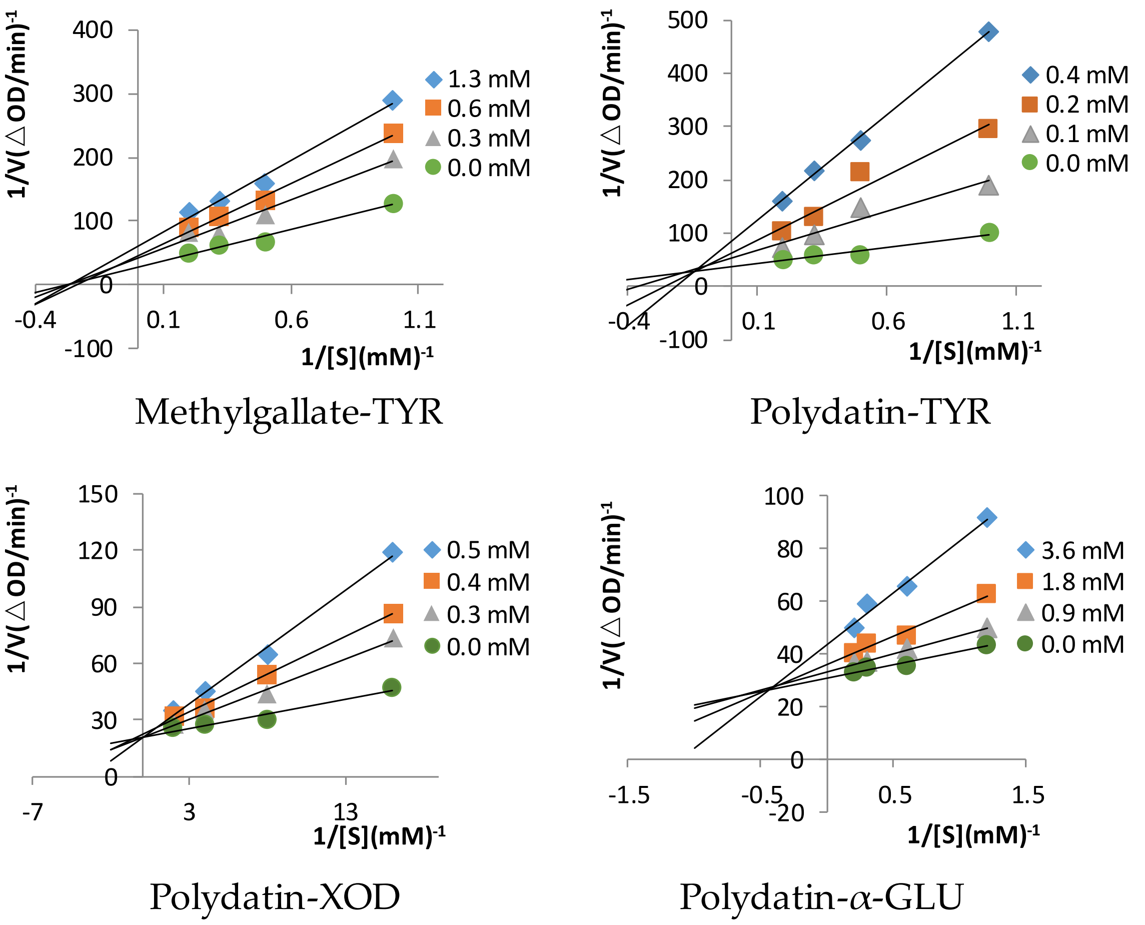


**Figure S2** Lineweaver-Burk plots of methylgallate and polydatin on the activity of three enzymes.
